# *SeqSQC*: A *Bioconductor* Package for Evaluating the Sample Quality of Next-generation Sequencing Data

**DOI:** 10.1016/j.gpb.2018.07.006

**Published:** 2019-04-05

**Authors:** Qian Liu, Qiang Hu, Song Yao, Marilyn L. Kwan, Janise M. Roh, Hua Zhao, Christine B. Ambrosone, Lawrence H. Kushi, Song Liu, Qianqian Zhu

**Affiliations:** 1 Department of Biostatistics, University at Buffalo, SUNY, Buffalo NY14260, USA; 2 Department of Biostatistics and Bioinformatics, Roswell Park Comprehensive Cancer Center, Buffalo NY14263, USA; 3 Department of Cancer Prevention and Control, Roswell Park Comprehensive Cancer Center, Buffalo NY14263, USA; 4 Division of Research, Kaiser Permanente Northern California, Oakland CA94612, USA; 5 Department of Epidemiology, The University of Texas MD Anderson Cancer Center, Houston TX77030, USA

**Keywords:** Next-generation sequencing, Quality assessment, 1000 Genomes Project, Whole-exome sequencing, *Bioconductor* package

## Abstract

As **next-generation sequencing** (NGS) technology has become widely used to identify genetic causal variants for various diseases and traits, a number of packages for checking NGS data quality have sprung up in public domains. In addition to the quality of sequencing data, sample quality issues, such as gender mismatch, abnormal inbreeding coefficient, cryptic relatedness, and population outliers, can also have fundamental impact on downstream analysis. However, there is a lack of tools specialized in identifying problematic samples from NGS data, often due to the limitation of sample size and variant counts. We developed *SeqSQC*, a ***Bioconductor* package**, to automate and accelerate sample cleaning in NGS data of any scale. *SeqSQC* is designed for efficient data storage and access, and equipped with interactive plots for intuitive data visualization to expedite the identification of problematic samples. *SeqSQC* is available at http://bioconductor.org/packages/SeqSQC.

## Introduction

The past several years have seen the explosion of genetic and genomic studies utilizing next-generation sequencing (NGS) technology in basic sciences, translational research, and clinics [1], [2], [3], [4], [5], [6], [7]. The high-throughput data generated from NGS bring new challenges to data processing, analysis, and interpretation [8]. A successful NGS study relies in large part on rigorous quality control (QC) to ensure that artifacts are removed before data analysis, so that real signals are not masked by quality issues. There are three levels of QC process: base/read level QC to clean up raw sequencing data; sample level QC to remove population outliers and problematic samples with gender mismatch, abnormal inbreeding coefficient, or cryptic relatedness; and variant level QC to eliminate inaccurate variant calls, for example, those resulting from sequencing errors in homo-polymers and incorrect read mapping.

Most currently available QC tools for NGS data are designed for the base/read level QC, which typically involves assessing the intrinsic quality of the raw reads to diagnose artifacts that arise from the library preparation and sequencing run [9], [10], [11], [12], [13], [14]. For instance, *NGSQC* [9] can monitor base/color code across each tile/panel, as well as quality measures for paired-end/mate pair libraries, whereas *NGS QC Toolkit* [10] is designed for homo-polymer trimming and primer/adaptor contamination removal. In addition, FastQC (https://www.bioinformatics.babraham.ac.uk/projects/fastqc/) provides comprehensive assessment of variation in quality scores and sequence content across the base/sequence/tile, sequence length distribution and duplication levels, as well as sequence over-representation. *QuaCRS* [13], an integrated quality control pipeline for RNA-Seq data, incorporates several R tools like *FastQC* for per-base read quality, *RNA-SeQC* for summarization of QC metric in a table format, and *RSeQC* [15] for useful saturation functions. QC-chain [14] is a tool for quality assessment and trimming of raw reads, identification, quantification, and filtration of unknown contamination.

In contrast, there is no publicly available tool designed to perform sample level QC on NGS data. Although the principles and steps for the sample level QC are essentially the same between NGS data and genome-wide association study (GWAS) data, there are new challenges inherent to the NGS that prevent us from directly using the tools designed for GWAS data, such as PLINK [16], *SNPRelate* [17], *GWASTools* [18], *GenABEL* [19], and *QCGWAS* [20]. First, unlike GWAS analyses, which usually include thousands of samples, NGS studies typically involve a much smaller sample size due to the still high cost of sequencing compared to genotyping. Second, while whole-exome sequencing (WES) is more cost-effective than whole-genome sequencing (WGS), the total number of variants generated from WES is much smaller, usually at the scale of around 250,000 for a sample size of 50. The calculations of metrics for sample level QC, such as sample relatedness, require large numbers of samples and variants to generate reliable estimates, which are not available for many NGS studies. For example, PLINK prefers at least 100,000 independent variants for estimating sample relatedness, which exceeds the number of linkage disequilibrium (LD)-pruned variants generated from typical WES studies of 50 samples (∼65,000 variants). Although PLINK/SEQ (https://atgu.mgh.harvard.edu/plinkseq/) allows variant summary and filtering, it is designed specifically for large-scale and population-based sequencing data, and unlike PLINK, it does not have a component for sample level QC.

Here, we present *SeqSQC*, a *Bioconductor* package, for sample level QC in NGS studies. *SeqSQC* takes variant calling format (VCF) files and sample annotation file containing sample population and gender information as input and reports problematic samples to be removed from downstream analysis. Through incorporation of benchmark data assembled from the 1000 Genomes Project, *SeqSQC* can accommodate NGS studies of small sample size and low number of variants.

## Method

### Assembly of benchmark dataset

We collected 87 samples from WGS data of the 1000 Genomes Project (Phase 3, release 20130502) as a benchmark dataset (Table 1), which includes 22 African (AFR) samples, 22 East Asian (EAS) samples, 21 European (EUR) samples, and 22 South Asian (SAS) samples. We selected 1–3 related pairs from each population that best represented the corresponding relationships (*e.g*., parent–offspring pairs, and full or half sibling pairs) and then randomly selected unrelated samples for a total of 20 pedigrees per population. As a result, there are eight known related pairs including four parent–offspring pairs, two full-sibling pairs, and two half-sibling or avuncular pairs in the benchmark dataset. The benchmark dataset contained only variants with minor allele frequency (MAF) >0.01 in at least one of the four populations. For a given NGS study cohort of interest, *SeqSQC* merges the benchmark dataset with the NGS dataset of the study cohort to form a final dataset for QC and only variants present in the benchmark dataset are used for sample level QC. For variants absent from the study cohort, a homozygous reference allele is assumed as long as the variants are located within the capture regions of the NGS platform employed.

**Table 1:** **Dataset from the 1000 Genomes Project**

**Dataset**	**Population**	**No. of samples**	**No. of related pairs**
Benchmark	AFR	22	3 (2 PO + 1 FS)
	EAS	22	2 (1 FS + 1 HF)
	EUR	21	1 (1 HF)
	SAS	22	2 (2 PO)

Test cohorts	AFR	647 AFR + 2 EAS + 2 EUR + 2 SAS + 1 DU + 1 CTM	6 (1 PO + 4 FS + 1 HF)
	EAS	493 EAS + 2 AFR + 2 EUR + 2 SAS + 1 DU + 1 CTM	9 (3 PO + 3 FS + 3 HF)
	EUR	484 EUR + 2 AFR + 2 EAS + 2 SAS + 1 DU + 1 CTM	1 (1 FS)
	SAS	472 SAS + 2 AFR + 2 EAS + 2 EUR + 1 DU + 1 CTM	3 (2 PO + 1 HF)

*Note*: PO, parent-offspring; FS, full sibling; HF, half sibling/avuncular pair; AFR, African; EAS, East Asian; EUR, European; SAS, South Asian; DU, duplicate; CTM, contamination.

### Test cohorts from the 1000 Genomes Project

To test the performance of *SeqSQC*, the remaining samples (after excluding those in the benchmark dataset) from the 1000 Genomes Project were grouped into four test cohorts according to the ancestries (647 AFR, 493 EAS, 484 EUR, and 472 SAS). We then added six random population outliers (two from each of the other three populations) to each test cohort. We also intentionally added one duplicate sample and one contaminated sample to each test cohort. The intended duplicate sample was a duplicate of one sample randomly selected from the test cohort, whereas the contaminated sample was generated by combining the genotypes from five randomly selected samples in the test cohort. All samples in each test cohort were summarized in Table 1. To mimic the WES data, we retained in the test cohorts only the variants located within the capture regions of Agilent SureSelect Human Exon v5, one of the most popular capture platforms to date.

To corroborate the results of *SeqSQC*, PLINK was also used to perform sample QC in each test cohort based on all the WGS variants that have MAF ≥0.01, missing rate ≤0.1, and did not violate the Hardy–Weinberg equilibrium (HWE) (*P* ≥ 1E−6). The variants were LD-pruned before the calculation of inbreeding coefficients and identity by descent (IBD) coefficients. For the sex check, a sample is predicted to be female or male if the X chromosome inbreeding coefficient is ≤0.2 or ≥0.8. For inbreeding check, samples with inbreeding coefficients that are five standard deviations beyond the mean are considered problematic. For IBD check, sample pairs with the proportion of IBD (PI_HAT) ≥0.125 are predicted as related.

To test the performance of *SeqSQC* on small sample size, we generated test cohorts consisting of one (HG00116), two (HG00116 and HG00120), or three samples (HG00116, HG00120, and NA18960). HG00116 is a male EUR, HG00120 is a female EUR and a relative of HG00116, whereas NA18960 is a male EAS and serves as an intended population outlier in the three-sample test cohort.

### Study cohorts of breast cancer WES data

We performed WES on 143 triple-negative breast cancer patients (all female) from three population groups (69 AFR, 26 Asian (ASN), and 48 EUR), using Agilent SureSelect Human Exon v5 capture kit. Specimens were obtained from the Pathways Study, a prospective cohort study of women diagnosed with breast cancer in the Kaiser Permanente Northern California health system [21], and from the Data Bank and BioRepository (DBBR) at Roswell Park Comprehensive Cancer Center [22] (126 and 17 samples, respectively). We applied *SeqSQC* to this dataset to examine the impact of sample QC on downstream analysis of breast cancer risk genes. When the population in the study cohort was specified as ASN, both EAS and SAS samples in the benchmark dataset were considered from the same population as the study cohort and were included for the sex check and inbreeding check. In the population outlier check for ASN, principle component analysis (PCA) prediction other than EAS or SAS was considered as population outlier.

In order to identify candidate breast cancer risk genes, we first isolated rare functional variants, and then restricted to recurrent genes in the cohort (genes that were mutated in at least two individuals). To obtain rare variants, we first removed non-clinically associated variants in dbSNP [23] (dbSNP129), and then excluded any variants that were present in the 1000 Genomes Project [24], [25] (ALL population, 2015 August release) and the Exome Sequencing Project (ESP; ESP6500siv2 all; http://evs.gs.washington.edu/EVS/) [26], as well as any variants with MAF >0.1% in Exome Aggregation Consortium (ExAC; exac03nontcga) [27]. We also filtered out variants that were not functionally important, including non-exonic variants (except splicing variants), synonymous variants, and nonsynonymous variants that are predicted to be benign by multiple bioinformatics software, including SIFT [28], PolyPhen2 [29], [30] (PolyPhen 2 HDIV, PolyPhen 2 HVar), LRT [31], MutationTaster [32], MutationAssessor [33], FATHMM [34], MetaSVM, and MetaLR [35]. Variants in segmental duplications were also excluded due to high false positive rate of variant calling [36]. ANNOVAR [37] was used to facilitate these variant filtering steps. We further filtered out long insertions and deletions (>20 bp) and any variants in genes that are not expressed in breast.

### Implementation

**Figure 1:**
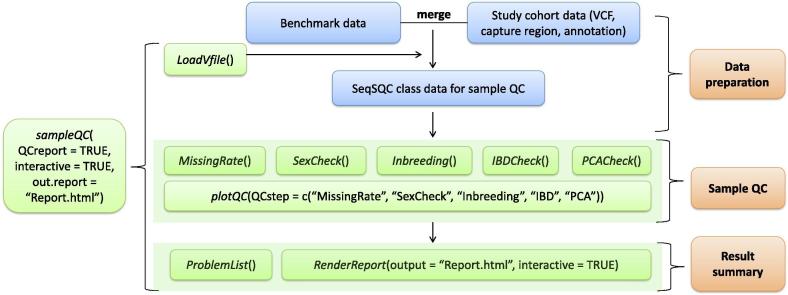
**Flowchart of the *SeqSQC* functionalities** In the data preparation module, *SeqSQC* merges the study cohort with the benchmark data. Merged data of *SeqSQC* class are used for the subsequent sample QC and result summary. The input files allowed in *SeqSQC* include a VCF file, a BED file for capture region, and an annotation file with sample population and gender information. User could use the wrap up function for an automated sample QC, to generate all QC results, a problematic sample list with indication of the reason for removal, and a sample QC report with interactive plots for each QC step. User can also call the specific QC function, or customize the settings of each QC step, including the criteria for defining problematic samples and the choice of statistical methods.

A flowchart of *SeqSQC* functionalities is displayed in Figure 1. *SeqSQC* consists of three major modules: data preparation, sample QC, and result summary. The sample QC module includes the following five steps: missing rate check, sex check, inbreedingcheck, IBD check, and population outlier check. The entire sample level QC is wrapped up in one function: *sampleQC*. By executing this function, a list of problematic samples and a QC report with interactive plots in html format, are generated according to the criteria defined for each QC step. Problematic samples identified at each QC step are automatically removed before getting to the next step. We provide a brief overview of *SeqSQC* as below. A more detailed description of package functionality and usage can be found in the package vignette and manual [*R* console type in *browseVignettes(“SeqSQC”)* for the vignette], or at the *Bioconductor* website for *SeqSQC*: (http://bioconductor.org/packages/SeqSQC).

#### Input

Only bi-allelic single nucleotide variants (SNVs) from the VCF input are included as input for sample QC analysis.

#### Sample missing rate check

Samples with a missing rate >0.1 are considered problematic. Functions *MissingRate and plotQC*(QCstep = “MissingRate”) are developed to calculate and plot the sample missing rate, respectively.

#### Sex check

We first filter out the pseudo-autosomal regions in X chromosome. Then the sample inbreeding coefficient (F) is calculated based on the numbers of variants on X chromosome for all samples in the study cohort and those for benchmark samples of the same population as the study cohort. The sample is predicted to be female with F≤0.2 and male with F≥0.8, while the samples with 0.2<F<0.8, are considered as ambiguous (pred.sex = 0). Accordingly, the sample gender is predicted using the function *SexCheck*, while the X chromosome inbreeding coefficients are plotted using *plotQC*(QCstep = “SexCheck”), where samples with gender mismatch are highlighted.

#### Inbreeding check

Using LD-pruned autosomal variants, we calculate the inbreeding coefficients for each sample in the study cohort and for benchmark samples of the same population as the study cohort. Samples with inbreeding coefficients that are five standard deviations beyond the mean are considered problematic. Functions *Inbreeding* and *plotQC*(QCstep = “Inbreeding”) are used to calculate and plot the inbreeding coefficients, respectively.

#### IBD check

Using LD-pruned autosomal variants, we first calculate the IBD coefficients for all sample pairs. We then predict related sample pairs in study cohort using the support vector machine (SVM) method [38] with linear kernel and the known relatedness embedded in benchmark data as the training set. All predicted related pairs are also required to have a coefficient of kinship ≥0.08. The sample with higher missing rate in each related pair is removed. The function *IBDCheck* calculates the IBD coefficients for each sample pair and predicts the relatedness for samples in the study cohort. The function *plotQC*(QCstep = “IBD”) then draws the descent coefficients, K0 and K1, for each pair.

#### Population outlier check

Using LD-pruned autosomal variants, we calculate the eigenvectors and eigenvalues for PCA. We use the benchmark samples as training dataset, and predict the population group for each sample in the study cohort using the top four eigenvectors and SVM with linear kernel. Samples with discordant predicted and self-reported population groups are considered problematic. The function *PCACheck* performs the PCA analysis and identifies population outliers in study cohort, whereas the function *plotQC*(QCstep = “PCA”) draws the eigenvectors of the first two PC axes for all samples by default.

## Results

One strength of *SeqSQC* is that it incorporates a benchmark dataset generated from the 1000 Genomes Project with the study cohort (the NGS data to be checked for quality) during the QC process. This benchmark dataset contains 20 independent samples selected from each of the four major populations (AFR, EAS, EUR, and SAS) and eight related sample pairs (4 parent–offspring pairs, 2 full-sibling pairs, and 2 half-sibling or avuncular pairs) (Table 1 and Methods). The benchmark serves as a supervised guide to the identification of problematic samples. It is especially useful for NGS data with limited sample size or variant number, as merging with the benchmark data could automatically boost the sample size and variant number for the study cohorts.

### Evaluation of *SeqSQC* performance using test cohorts from the 1000 Genomes Project

**Figure 2:**
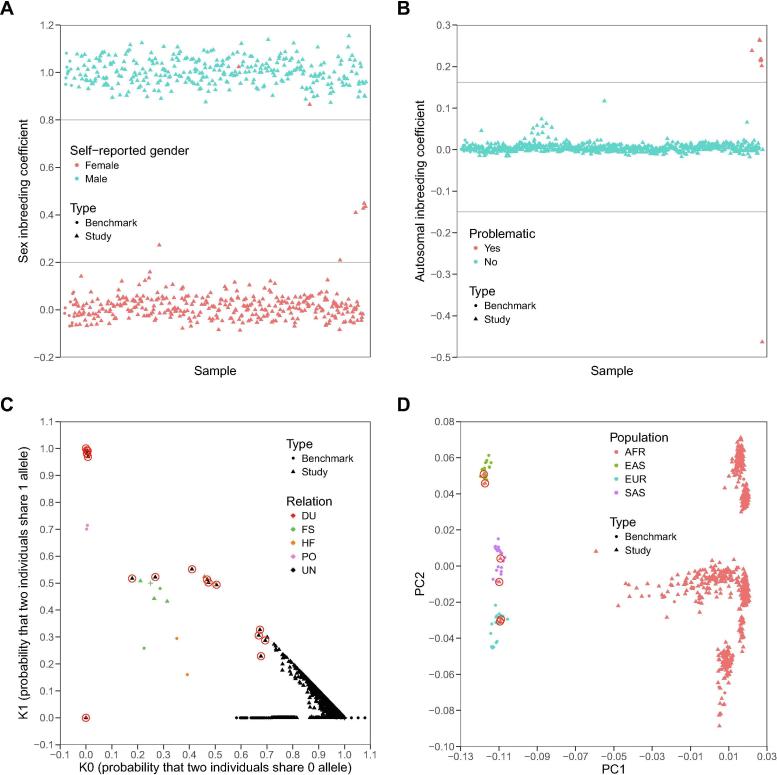
**The sample quality check for the AFR test cohort from the 1000 Genomes Project** **A.** Sex check. 655 study samples and 22 benchmark samples of AFR ancestry were shown. Gray lines were drawn when sex inbreeding coefficient equals 0.2 or 0.8 as threshold for sample genders (See Method). Two self-reported female samples were detected to be male by *SeqSQC* (indicated as two red triangles among the group of cyan triangles). **B.** The plot of inbreeding coefficients. 655 study samples and 22 benchmark samples of AFR ancestry were shown. Gray lines were drawn when autosomal inbreeding coefficient equals to five standard deviations beyond mean. Any point beyond the gray lines was defined to be problematic. Eight inbreeding outliers were detected (including one simulated sample with contamination, six intended population outliers, and one unintended inbreeding outlier; see Tables S1 and S2). **C.** IBD check. After removing problematic samples detected from previous QC steps, a total of 732 samples (including 645 study samples and 87 benchmark samples) were shown in pairwise fashion. Samples with known relationships are highlighted, including DU (red), FS (green), HF (organge), and PO (pink), whereas samples with unknown relationship were marked in black. “+” highlights the expected position for each corresponding relationship. Newly-detected relationships from this test cohort are highlighted with red circles. **D.** The plot of the first two PC axes from the PCA analysis. After removing problematic samples detected from previous QC steps except for the six intended population outliers, as well as the related samples in benchmark data, a total of 718 independent samples (including 638 study samples and 80 benchmark samples) were shown. Six intended population outliers (two from each population of EAS, EUR, and SAS) are highlighted with red circles. The AFR samples were separated into different groups in PC2 since they came from different sub-populations including ACB, ASW, ESN, GWD, LWK, MSL, and YRI. AFR, African; EAS, East Asian; EUR, European; SAS, South Asian; DU, duplicate; FS, full-sibling; HF, half-sibling/avuncular pair; UN, unknown; PO, parent–offspring pair; PCA, principal component analysis; ACB, African Caribbeans in Barbados; ASW, Americans of African ancestry in Southwestern USA; ESN, Esan in Nigeria; GWD, Gambian in Western Divisions in the Gambia; LWK, Luhya in Webuye, Kenya; MSL, Mende in Sierra Leone; YRI, Yoruba in Ibadan, Nigeria.

In order to evaluate the performance of *SeqSQC* in identifying problematic samples, we generated four test cohorts from the 1000 Genomes Project for each of the four major populations (AFR, EAS, EUR, and SAS) as the true identity of these samples is known. In each test cohort, we embedded one intended duplicate sample, one contrived contaminated sample, and six population outliers (Table 1 and Methods). Since samples from the 1000 Genome Project were whole-genome sequenced, to mimic WES data, we kept in the VCF file only those variants that fall in capture regions of Agilent SureSelect Human Exon v5 platform (see Method section). As expected, *SeqSQC* successfully detected the contaminated sample in inbreeding check, the duplicate sample in IBD check, and all six population outliers in either inbreeding check or population outlier check (Table S1 and Figure 2). There were a total of 19 self-reported related pairs in the four test cohorts. *SeqSQC* confirmed 18 of them but identified one self-reported full-sibling pair in the AFR test cohort as unrelated. Notably, this full-sibling pair was confirmed to be unrelated using the IBD segment sharing analysis from the 1000 Genomes Project.

**Supplementary Figure S1:**
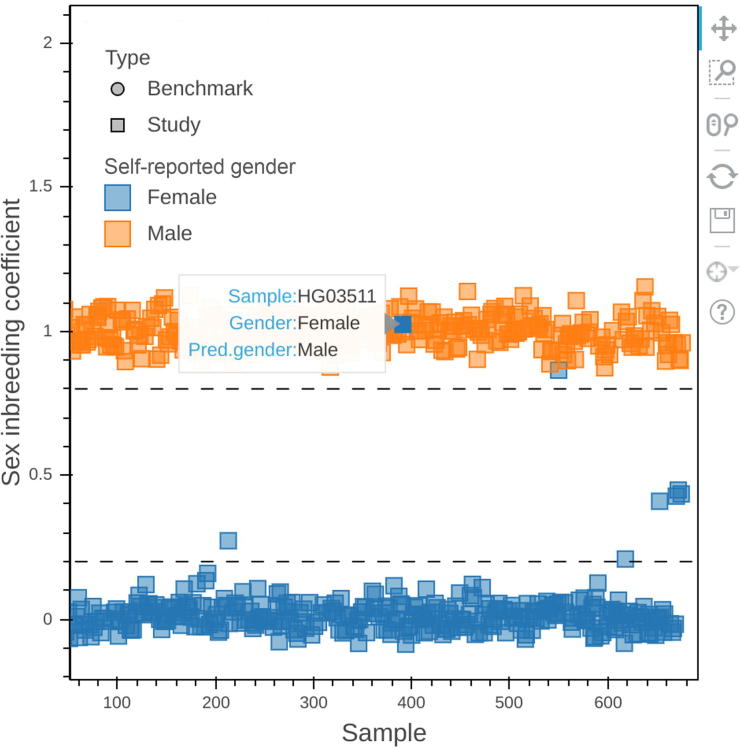
**The screenshot of interactive plot of the sex check result for the AFR test cohort from the 1000 Genomes Project** One of the two samples with gender mismatch was highlighted in this interactive plot. This self-reported female sample was detected as male by *SeqSQC*.

Surprisingly, *SeqSQC* also detected additional unintended problematic samples in each of the test cohorts (Table S1). In the AFR test cohort, two self-reported female samples were predicted to be male by *SeqSQC* (Figure 2A and Figure S1), in addition to one inbreeding outlier (Figure 2B) and 12 related sample pairs detected (Figure 2C). Moreover, *SeqSQC* identified three, two, and six related sample pairs in the EAS, EUR, and SAS test cohorts, respectively, and another two samples with gender mismatch identified in the EUR test cohort.

As an alternative approach to corroborate these new problematic samples identified by *SeqSQC*, we used PLINK to carry out sample QC based on the entire WGS data of the same samples, which are more than 30 times larger than the data used by *SeqSQC* (Methods). PLINK confirmed all the newly identified problematic samples by *SeqSQC*, including the four gender mismatch samples, 23 related samples, and one inbreeding outlier. The list of these problematic samples (or sample pairs) is provided in Table S2.

To demonstrate the capability of *SeqSQC* to perform sample QC on NGS data with small sample size, we generated test cohorts with only one, two, or three samples from the 1000 Genomes Project, respectively. As shown in Figure S2, *SeqSQC* correctly identified the sample characteristics and pinpointed problematic samples on these small datasets.

### Application of *SeqSQC* to study cohorts of breast cancer WES data

We showed here an example of *SeqSQC* application to the “real-world” WES data. This WES dataset contained 143 triple-negative breast cancer patients from three populations (69 AFR, 26 ASN, and 48 EUR). *SeqSQC* was run on each population for sample-level QC.

*SeqSQC* detected two inbreeding outliers (one AFR and one EUR), and four population outliers (two samples each from AFR and ASN populations) (Table 2, Figures S3 and S4). After removing these six problematic samples, the numbers of recurrent genes as well as the contained rare and potentially functional variants were reduced from 1887 to 1803 and from 4643 to 4436, respectively. These data indicate that sample-level QC has non-trivial impact on downstream analysis of breast cancer risk genes.

**Table 2:** **The problematic samples in WES of 143 breast cancer patients**

**Population**	**No. of study samples**	**No. of problematic samples**	**Reason for removal**
AFR	69	1	Inbreeding outlier
		2	Population outlier

EUR	48	1	Inbreeding outlier

ASN	26	2	Population outlier

## Conclusion

*SeqSQC* is a *Bioconductor* package that automates and accelerates sample cleaning of NGS data on any scale. It enables the identification of problematic samples with high missing rate, gender mismatch, contamination, abnormal inbreeding coefficient, cryptic relatedness, or discordant population information. With a built-in benchmark dataset carefully assembled from the 1000 Genomes Project, *SeqSQC* is particularly useful for NGS studies with limited sample size or variant number. Designed with efficiency in mind, it stores the genotype in Genomic Data Structure (GDS) format, which could increase the data storage efficiency by 5-fold and data access speed by 2–3-fold, respectively [18], [39]. For example, it took less than 10 min to complete all sample QC steps for 143 WES samples from the study cohort of breast cancer patients (32 Gb main memory, 2.00 GHz Intel® Xeon® E5-2620). *SeqSQC* is user-friendly in that the entire QC process is highly automated and only one command line is needed to get the final QC reports. The package generates interactive plots for each QC step as an intuitive interface for visualization. Furthermore, users can customize settings for the QC process, including the criteria for defining problematic samples and the choice of statistical methods.

Based on the WES variants of test cohorts assembled from the 1000 Genomes Project, *SeqSQC* successfully identified all intended problematic samples including the related samples, simulated contaminated sample, the duplicate sample, and the population outliers. *SeqSQC* also detected additional unexpected problematic samples. All these problematic samples were confirmed by PLINK when running on the same samples using WGS variants provided by the 1000 Genomes Project. Since the 1000 Genomes Project dataset is widely used around the world in genetic studies, a catalog of the problematic samples, such as those detected by *SeqSQC*, would be a useful resource to the research community.

We foresee a variety of extensions of *SeqSQC*. For example, due to insufficient first cousin pairs from the 1000 Genomes Project, the current version of *SeqSQC* does not aim to detect weak relatedness such as first cousins. With the continuous expansion of the 1000 Genomes Project and other publicly available sequencing projects, we will boost the sensitivity of detecting weak relationship by *SeqSQC* using upgraded benchmark data. Another issue that needs attention is how to handle sample QC in admixed population. Currently we only include the four most-studied population groups in the benchmark dataset (AFR, EUR, EAS, and SAS) in *SeqSQC*. The admixed population such as Hispanic or admixed-American could not be properly handled by *SeqSQC* yet. We expect that future inclusion of representative samples from admixed populations into the benchmark data could help bridge this gap. As potential batch effect could exist between the study dataset and the benchmark dataset, we will include a batch effect detection function in the future release of *SeqSQC*.

We recognize that sample QC can also be done before sequencing using either high-density SNP arrays or custom designed SNP panels (*e.g.*, iPLEX® Pro Sample ID Panel) to verify sample quality, gender, and relationships. As it allows picking up problematic samples before the expensive sequencing procedure, pre-sequencing sample QC is a good practice even though it will increase the cost and the DNA amount needed for the project. On the other hand even if samples are perfectly fine according to the pre-sequencing QC, technical errors like sample mislabeling and contamination can still happen during the library preparation and sequencing procedure, and therefore sample QC after sequencing is still necessary.

## Availability of data and materials

The datasets generated and/or analyzed in the current study are available upon request from the corresponding authors.

## Authors’ contributions

QL, QH, and QZ conceived the idea and designed the study. QL developed the software. QH, SY, MLK, JMR, LHK, HZ, CBA, and SL were involved in data interpretation. QL and QZ drafted the manuscript with the assistance of QH, SY, MLK, JMR, LHK, HZ, CBA, and SL. All authors read and approved the final manuscript.

## Competing interests

The authors have declared no competing interests.

## Supplementary data

Supplementary Figure S2 *The sample QC results for small sample size test cohorts from the 1000 Genomes Project* The sample QC results for the one-sample test cohort contained HG00116, which was reported to be a male EUR (**A**–**C**). **A.** Sex check. The sample was correctly identified by *SeqSQC* as a male. **B.** The plot of inbreeding coefficients for the one-sample test cohort. **C.** The plot of the first two PC axes for the one-sample test cohort. The sample was successfully identified as EUR. **D.** The two-sample test cohort contained HG00116 and HG00120, which were self-reported to be half-sibling but more likely first cousin as identified by *SeqSQC* and confirmed by *SeqSQC* using the test cohort of all EUR. “+” highlights the expected position for each corresponding relationship. The red circle highlights the sample pair of HG00116 and HG00120. **E.** The three-sample test cohort contained HG00116, HG00120, and NA18960, where NA18960 is a male from EAS and served as an intended population outlier. *SeqSQC* correctly identified NA18960 as a population outlier (highlighted by red circle).

Supplementary Figure S3 **The plot of inbreeding coefficients for the AFR and EUR samples from the breast cancer WES data A.** A total of 91 AFR samples (including 69 study samples and 22 benchmark samples) were plotted. One inbreeding outlier (red) was detected, which was defined to be five standard deviations beyond the mean. **B.** A total of 69 EUR samples (including 48 study samples and 21 benchmark samples) were plotted. One inbreeding outlier was detected, which was defined to be five standard deviations beyond the mean (red).

Supplementary Figure S4 **The plot of the first two PC axes for the ASN and AFR samples from the breast cancer WES data A.** A total of 106 samples (including 26 study samples of ASN ancestry and all 80 independent benchmark samples) were plotted. Two self-reported ASN samples were detected as population outliers (red circles). **B.** A total of 148 samples (including 68 study samples of AFR ancestry and all 80 independent benchmark samples) were plotted. Two self-reported AFR samples were detected as population outliers (red circles).

Supplementary Table S1 **Performance of SeqSQC in the test cohorts from the 1000 Genomes Project**

Supplementary Table S2 **List of unintended problematic samples in the 1000 Genomes Project detected by *SeqSQC***
