# Supplementary figures and images for: SeqSQC: A Bioconductor Package for Evaluating the Sample Quality of Next-generation Sequencing Data
Source: Genomics Proteomics Bioinformatics. 2019 Apr 5;17(2):211–8. doi: 10.1016/j.gpb.2018.07.006 (PMC6620264; doi:10.1016/j.gpb.2018.07.006)

**A**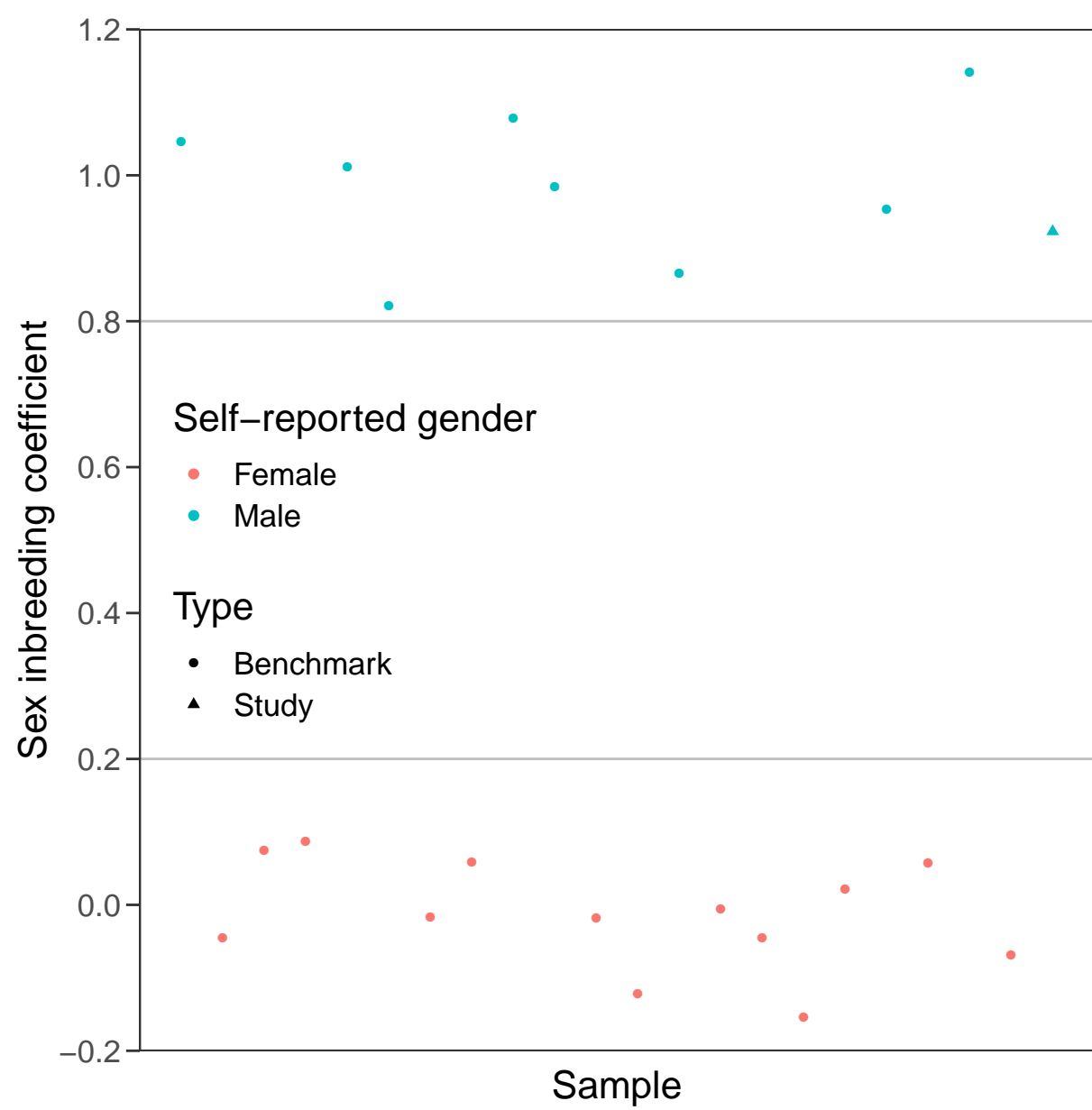**B**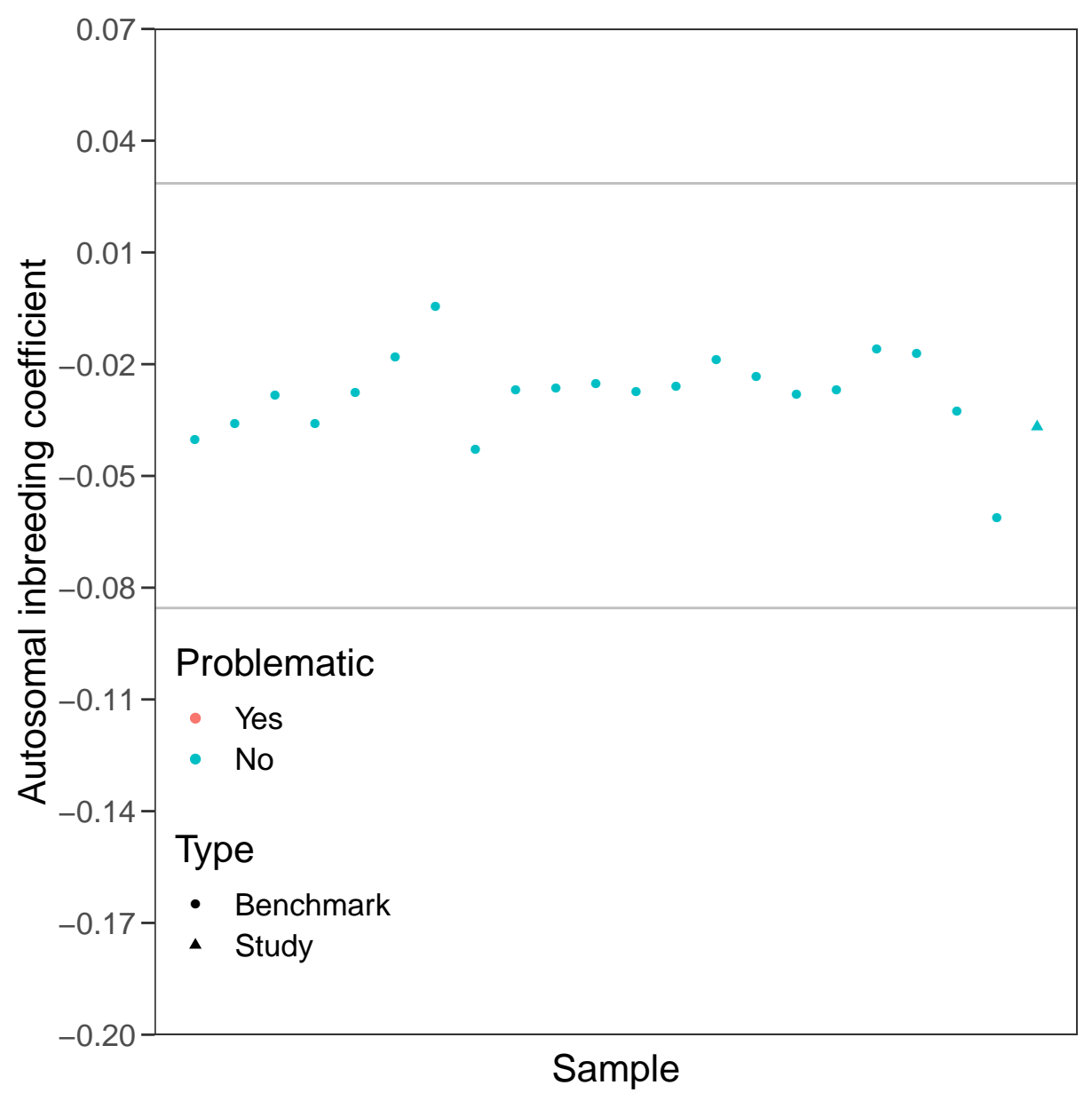**C**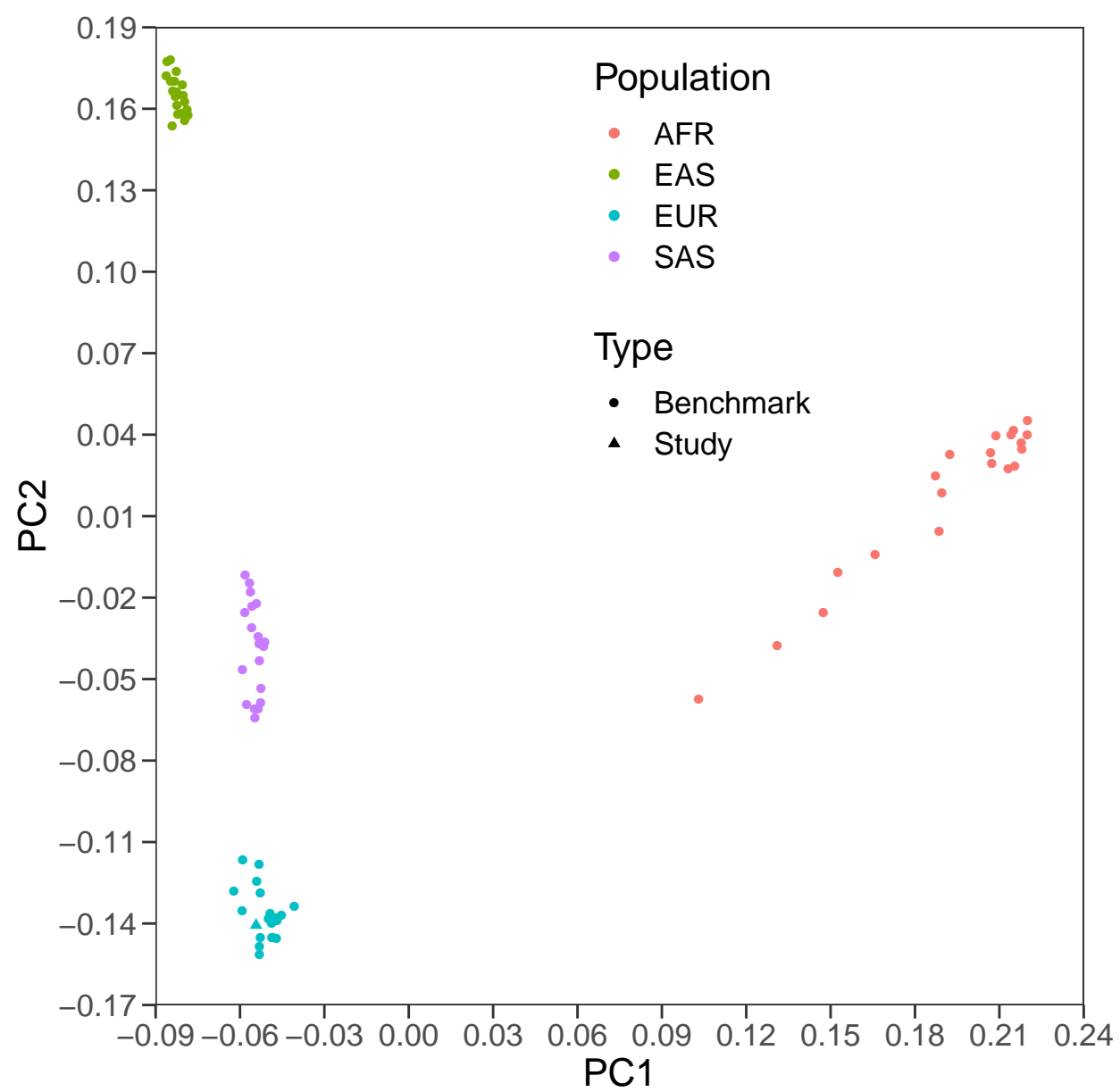**D**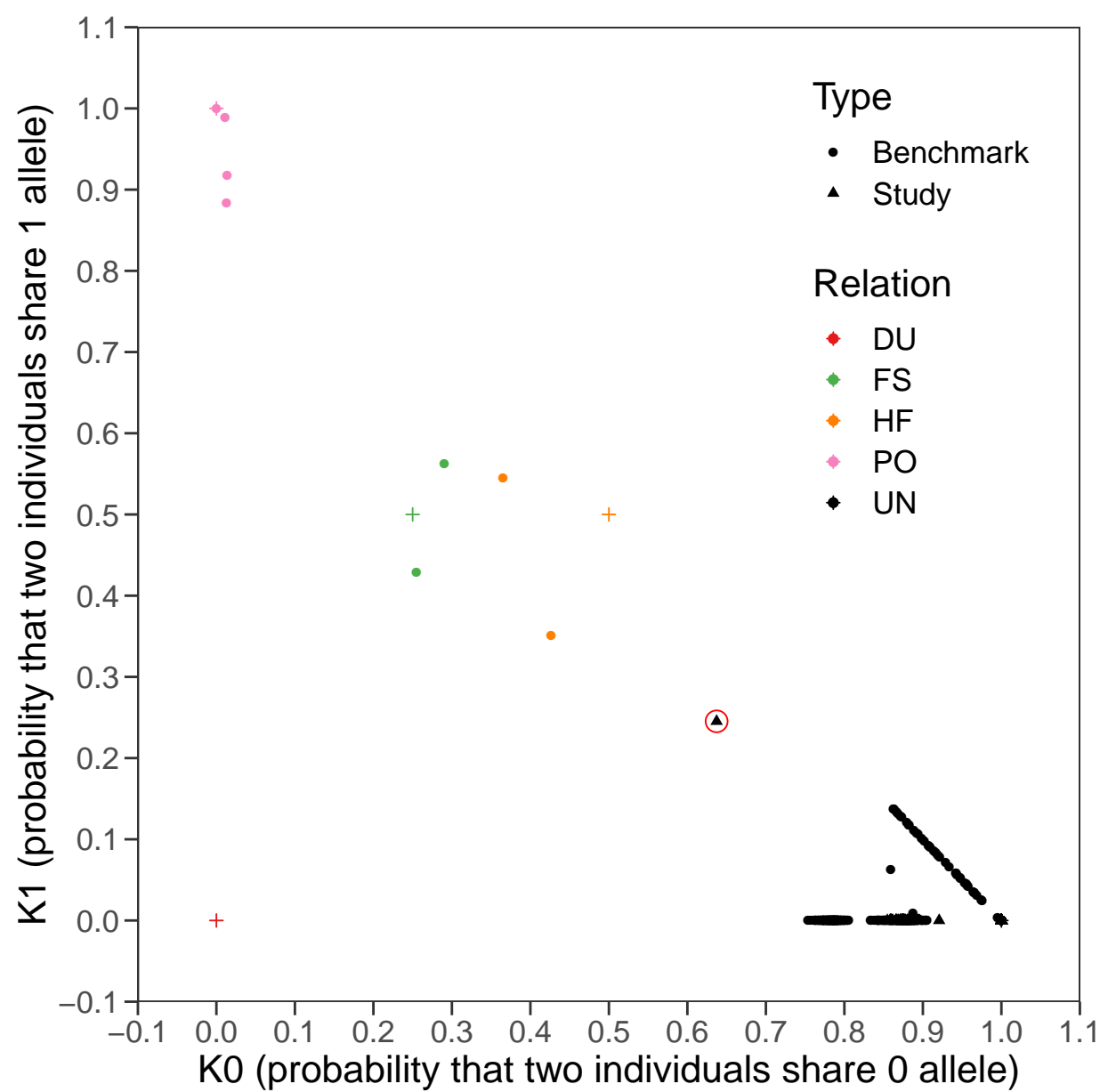**E**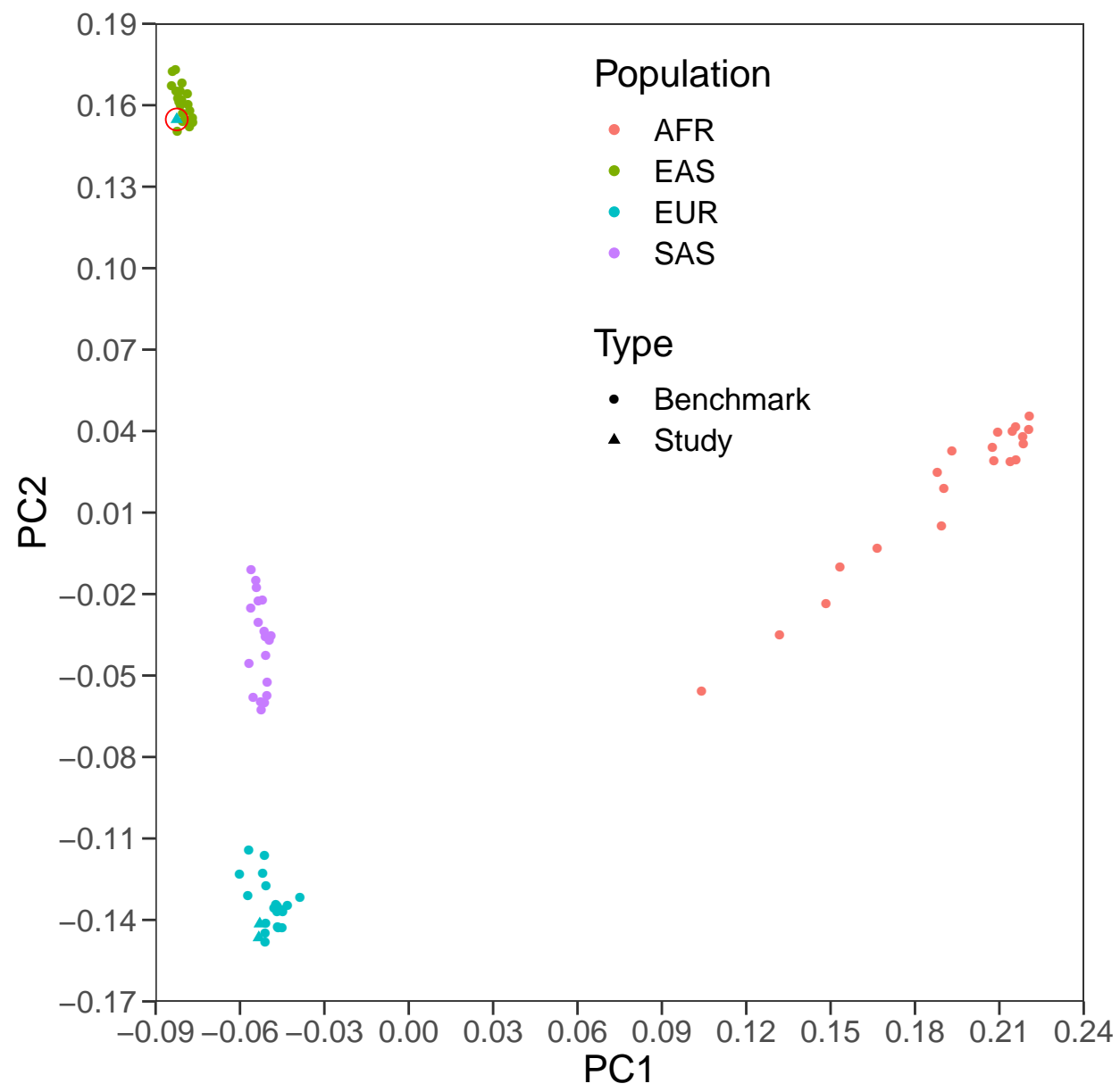

Supplement: Supplementary Figure S2 — The sample QC results for small sample size test cohorts from the 1000 Genomes Project The sample QC results for the one-sample test cohort contained HG00116, which was reported to be a male EUR (A–C). A. Sex check. The sample was correctly identified by SeqSQC as a male. B. The plot of inbreeding coefficients for the one-sample test cohort. C. The plot of the first two PC axes for the one-sample test cohort. The sample was successfully identified as EUR. D. The two-sample test cohort contained HG00116 and HG00120, which were self-reported to be half-sibling but more likely first cousin as identified by SeqSQC and confirmed by SeqSQC using the test cohort of all EUR. “+” highlights the expected position for each corresponding relationship. The red circle highlights the sample pair of HG00116 and HG00120. E. The three-sample test cohort contained HG00116, HG00120, and NA18960, where NA18960 is a male from EAS and served as an intended population outlier. SeqSQC correctly identified NA18960 as a population outlier (highlighted by red circle). [file mmc1.pdf]

**A**

Inbreeding coefficients for AFR

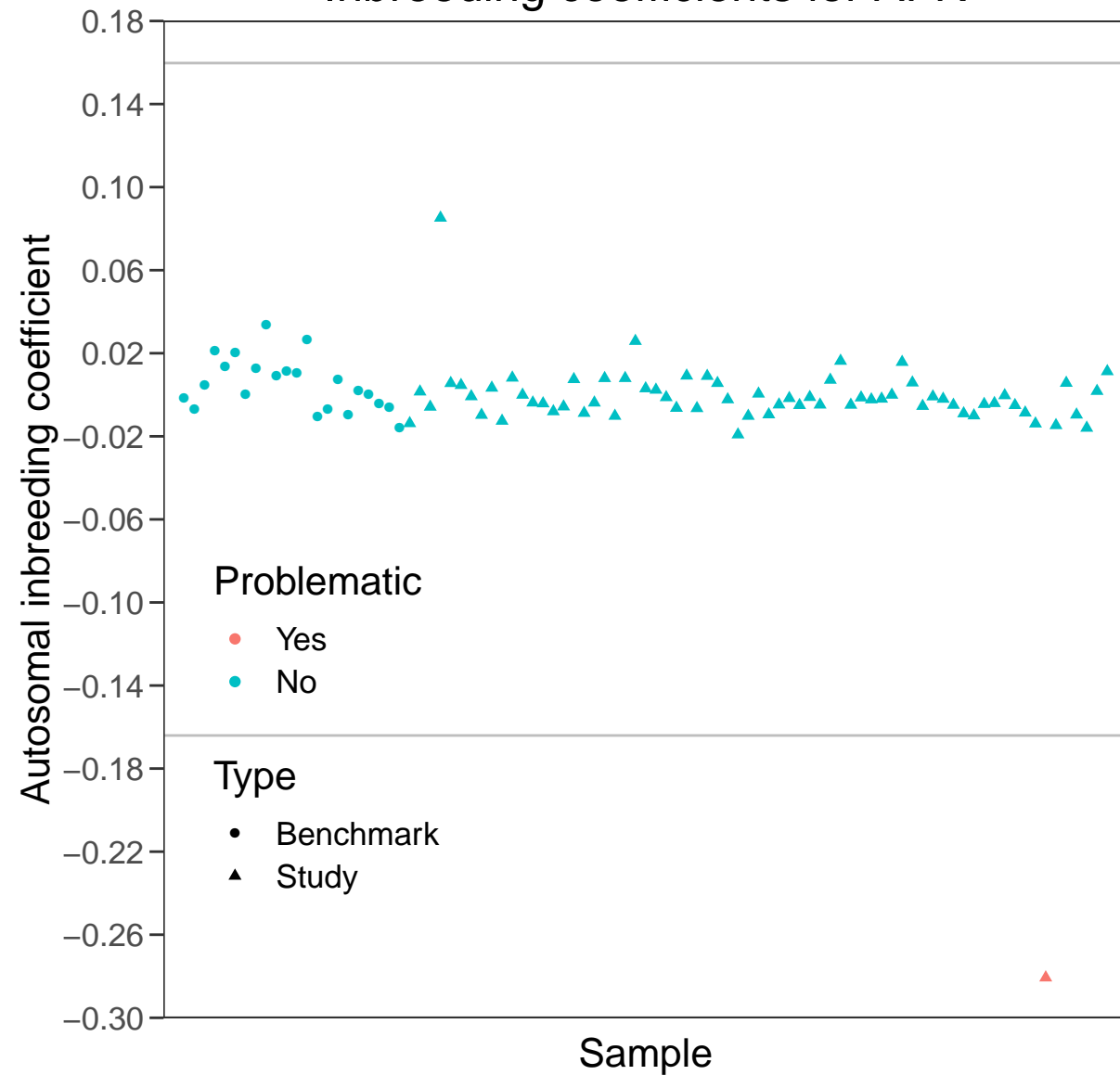**B**

Inbreeding coefficients for EUR

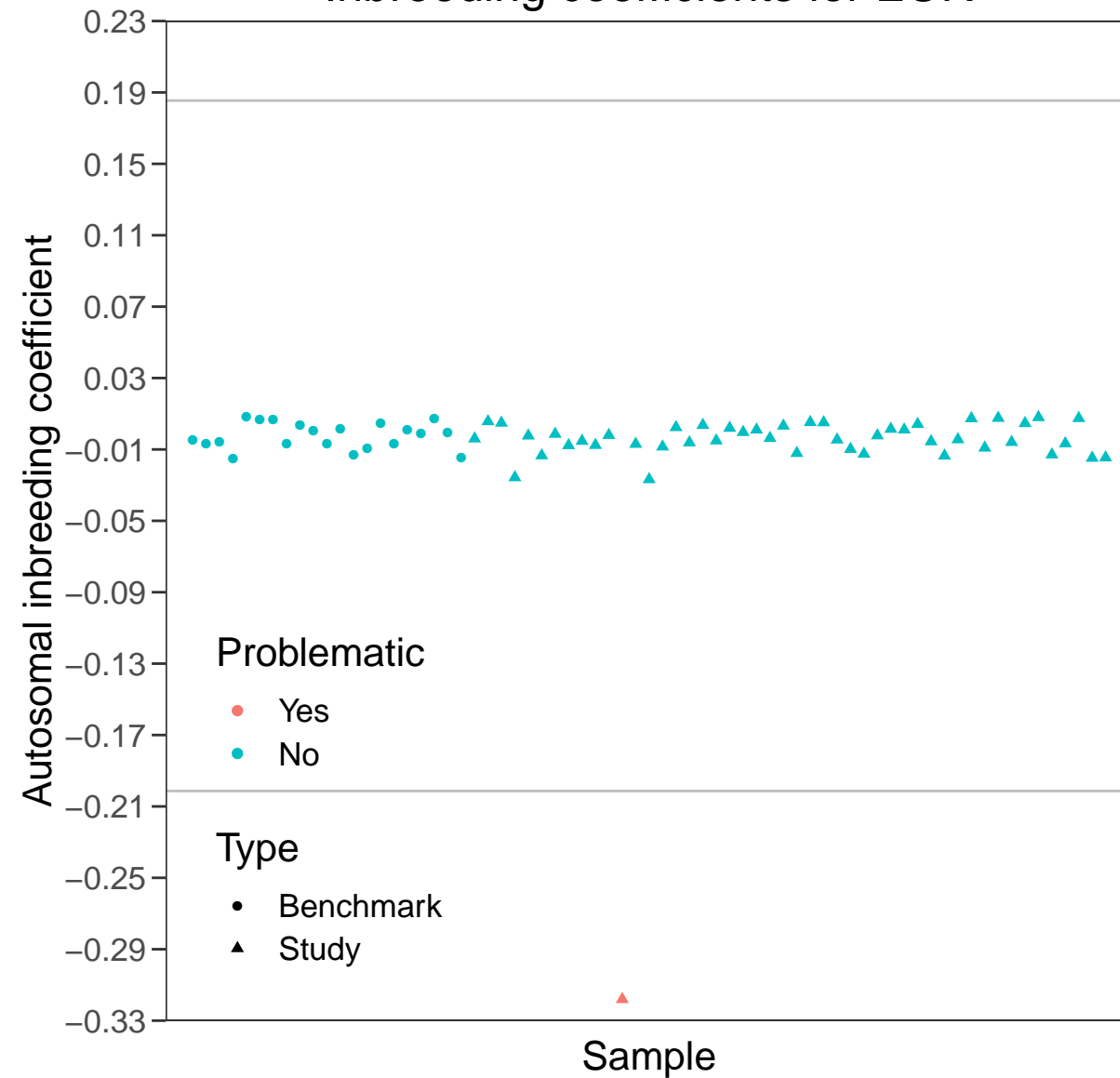

Supplement: Supplementary Figure S3 — The plot of inbreeding coefficients for the AFR and EUR samples from the breast cancer WES data A. A total of 91 AFR samples (including 69 study samples and 22 benchmark samples) were plotted. One inbreeding outlier (red) was detected, which was defined to be five standard deviations beyond the mean. B. A total of 69 EUR samples (including 48 study samples and 21 benchmark samples) were plotted. One inbreeding outlier was detected, which was defined to be five standard deviations beyond the mean (red). [file mmc2.pdf]

**A**

PC axes for ASN

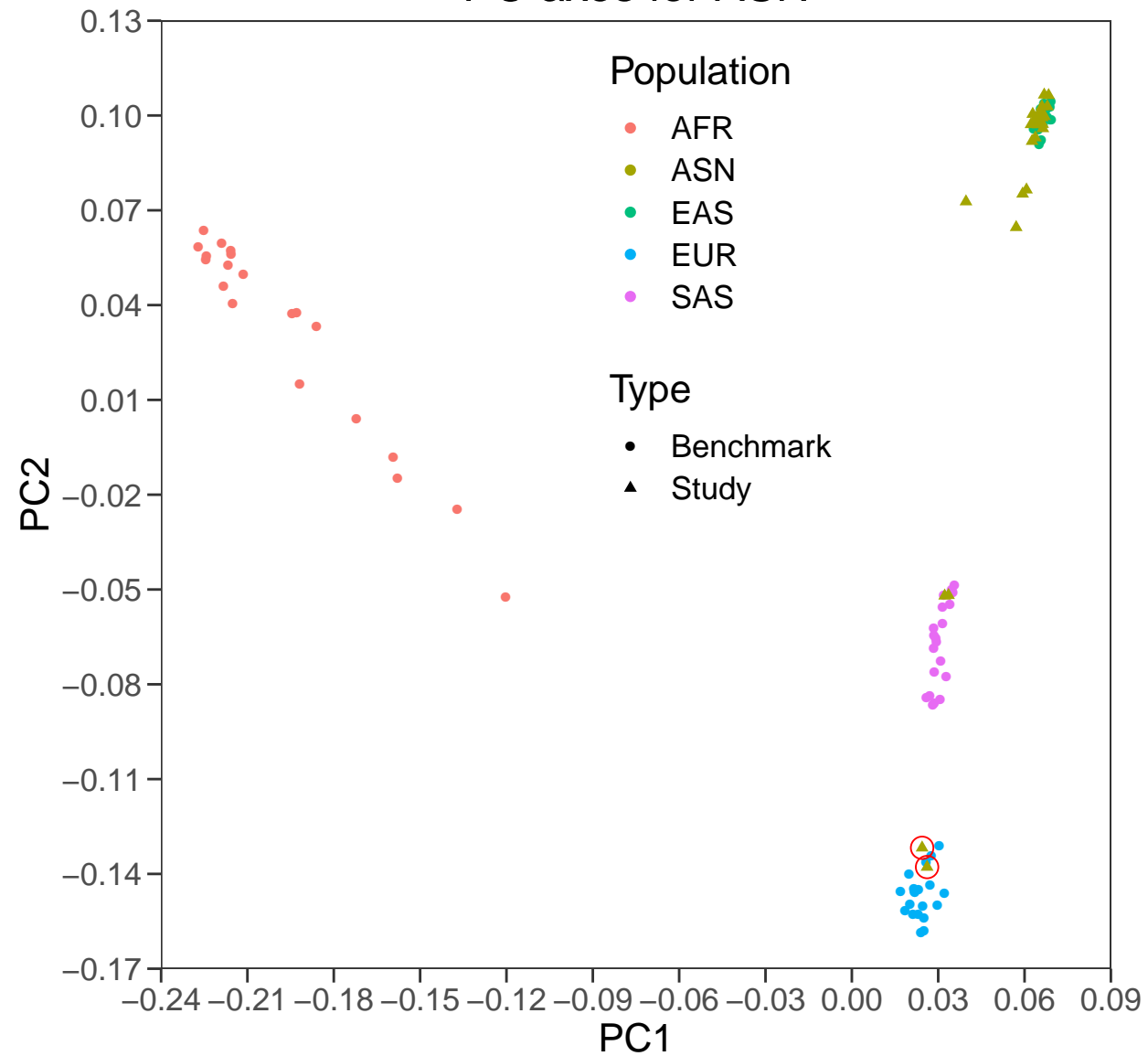**B**

PC axes for AFR

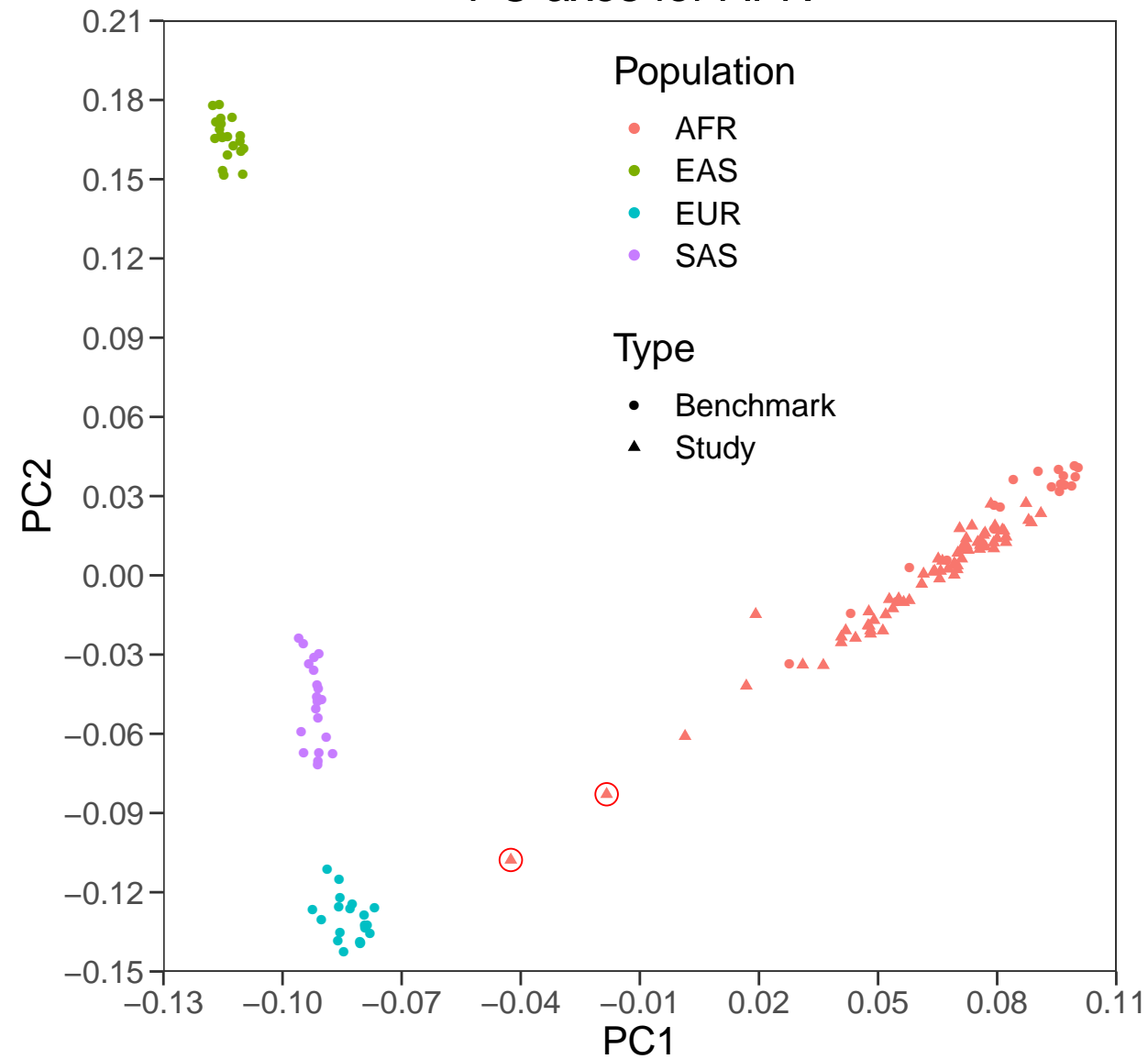

Supplement: Supplementary Figure S4 — The plot of the first two PC axes for the ASN and AFR samples from the breast cancer WES data A. A total of 106 samples (including 26 study samples of ASN ancestry and all 80 independent benchmark samples) were plotted. Two self-reported ASN samples were detected as population outliers (red circles). B. A total of 148 samples (including 68 study samples of AFR ancestry and all 80 independent benchmark samples) were plotted. Two self-reported AFR samples were detected as population outliers (red circles). [file mmc3.pdf]
