# Supplementary material for: SeqSQC: A Bioconductor Package for Evaluating the Sample Quality of Next-generation Sequencing Data
Source: Genomics Proteomics Bioinformatics. 2019 Apr 5;17(2):211–8. doi: 10.1016/j.gpb.2018.07.006 (PMC6620264; doi:10.1016/j.gpb.2018.07.006)
Supplement: Supplementary Table S1 — Performance of SeqSQC in the test cohorts from the 1000 Genomes Project [file mmc4.docx]

**Table S1 Performance of *SeqSQC* in the test cohorts from the 1000 Genomes Project**

| **Problem type** | **QC problem** | **Detection step in *SeqSQC*** | **No. of known problematic samples or related pairs**^‡^ | **No. of problematic samples or related pairs detected in *SeqSQC*** | | | |
| --- | --- | --- | --- | --- | --- | --- | --- |
|  |  |  |  | **AFR** | **EAS** | **EUR** | **SAS** |
| Intended^*^ | Contamination | Inbreeding check | 4 (1/1/1/1) | 1 | 1 | 1 | 1 |
|  | Duplicate | IBD check | 4 (1/1/1/1) | 1 | 1 | 1 | 1 |
|  | Relatedness | IBD check | 19 (6/9/1/3) | 5^§^ | 9 | 1 | 3 |
|  | Population outlier | Inbreeding / population outlier check | 24 (6/6/6/6) | 6 (6/0)^¥^ | 6 (4/2) | 6 (4/2) | 6 (2/4) |
| Unintended^†^ | Gender mismatch | Sex check |  | 2 | 0 | 2 | 0 |
|  | Inbreeding outlier | Inbreeding check |  | 1 | 0 | 0 | 0 |
|  | Relatedness | IBD check |  | 12 | 3 | 2 | 6 |

*Note*: ^*^, One simulated contaminated sample, one duplicate sample, and six population outliers were added in each test cohort (see Methods). Each test cohort also included samples that were self-reported relatives. ^†^, additional problematic samples detected by *SeqSQC* that were unknown to be problematic previously. ^‡^, total number and the number of problematic samples in each test cohort (AFR/EAS/EUR/SAS) were given. ^§^, One of the six intended related pairs was not detected in AFR test cohort. IBD segment sharing analysis in 1000 Genomes Project confirmed this pair to be unrelated (see Results). ^¥^, the numbers inside the parentheses were the number of known population outliers that *SeqSQC* detected in inbreeding check and population outlier check, respectively.
