# Supplementary material for: SeqSQC: A Bioconductor Package for Evaluating the Sample Quality of Next-generation Sequencing Data
Source: Genomics Proteomics Bioinformatics. 2019 Apr 5;17(2):211–8. doi: 10.1016/j.gpb.2018.07.006 (PMC6620264; doi:10.1016/j.gpb.2018.07.006)
Supplement: Supplementary Table S2 — List of unintended problematic samples in the 1000 Genomes Project detected by SeqSQC [file mmc5.docx]

**Table S2 List of unintended problematic samples in the 1000 Genomes Project detected by *SeqSQC***

| **Sample(s)** | **Reason for removal** | **Population** |
| --- | --- | --- |
| HG03511 | Gender mismatch | AFR |
| NA19332 | Gender mismatch | AFR |
| NA20314 | Inbreeding outlier | AFR |
| HG02429:HG02479 | Cryptic relationship | AFR |
| HG03343:HG03352 | Cryptic relationship | AFR |
| HG03464:HG03484 | Cryptic relationship | AFR |
| NA19313:NA19334 | Cryptic relationship | AFR |
| NA19025:NA19384 | Cryptic relationship | AFR |
| NA19904:NA19913 | Cryptic relationship | AFR |
| NA19625:NA20274 | Cryptic relationship | AFR |
| NA20274:NA20299 | Cryptic relationship | AFR |
| NA20317:NA20318 | Cryptic relationship | AFR |
| NA20322:NA20320 | Cryptic relationship | AFR |
| NA20336:NA20355 | Cryptic relationship | AFR |
| NA20359:NA20362 | Cryptic relationship | AFR |
| HG00475:HG00542 | Cryptic relationship | EAS |
| HG00702:HG00656 | Cryptic relationship | EAS |
| HG00702:HG00657 | Cryptic relationship | EAS |
| NA20506 | Gender mismatch | EUR |
| NA20530 | Gender mismatch | EUR |
| HG00116:HG00120 | Cryptic relationship | EUR |
| HG00238:HG00240 | Cryptic relationship | EUR |
| HG03750:HG03754 | Cryptic relationship | SAS |
| HG03733:HG03899 | Cryptic relationship | SAS |
| HG03873:HG03998 | Cryptic relationship | SAS |
| NA20882:NA20900 | Cryptic relationship | SAS |
| NA20891:NA20900 | Cryptic relationship | SAS |
| NA21109:NA21135 | Cryptic relationship | SAS |

*Note*: AFR, African; EAS, East Asian; EUR, European; SAS, South Asian.
